# Myocardial Infarction in Neonates: A Diagnostic and Therapeutic Challenge

**DOI:** 10.1155/2019/7203407

**Published:** 2019-10-24

**Authors:** Manuel Rodríguez Martínez, Eladio Ruiz González, Anna Parra-Llorca, Máximo Vento Torres, Marta Aguar Carrascosa

**Affiliations:** ^1^University and Polytechnic Hospital La Fe, Valencia, Spain; ^2^Division of Cardiology, University and Polytechnic Hospital La Fe, Valencia, Spain; ^3^Neonatal Research Group, Health Research Institute La Fe, Valencia, Spain; ^4^Division of Neonatology, University and Polytechnic Hospital La Fe, Valencia, Spain

## Abstract

Neonatal acute myocardial infarction is an uncommon entity. We describe the case of a 4-day-old term baby who presented with respiratory distress and distal acrocyanosis. The chest radiograph demonstrated cardiomegaly without pleural effusion, and examination revealed hepatomegaly. An electrocardiogram revealed QS pattern in leads I, aVL, and V6, suggestive of ischemia. Cardiac enzymes were elevated, and echocardiogram revealed moderate left ventricular dysfunction with a thrombus at the level of the left atrial appendage. The patient required hemodynamic stabilization, vasodilatation to avoid congestive heart failure, and anticoagulation with heparin and aspirin. In the context of this unusual diagnosis, we reviewed our experience over the last 17 years as well as the existing literature on neonatal myocardial infarction.

## 1. Introduction

Myocardial infarction (MI) is a common entity in adult population, mainly due to coronary artery disease. However, acute MI presenting in the neonatal period is rare, and the true incidence is still unknown due to limited reporting and diagnostic challenges. Multiple different etiologies have been suggested, but in many cases, the primary cause remains unknown. In cases in which a cause has been identified, culprits have included enteroviral myocarditis, eosinophilic endomyocarditis, congenital diaphragmatic hernia, coagulopathy, erythroblastosis, perinatal asphyxia, coronary artery thromboembolism caused by umbilical vein catheterization, obstructive congenital heart disease, intrauterine infection, and coronary artery vasoconstriction secondary to oxytocin administration [1–5]. MI is associated with poor prognosis [6], with a mortality rate ranging from 40 to 50%, according to different series [1, 7]. There are no specific clinical guidelines for appropriate management. Initial survival has improved markedly with recent treatment advances [8], including diuretics, angiotensin-converting enzyme inhibitors, inotropes, and in selected cases, thrombolysis and extracorporeal membrane oxygenation (ECMO) support [6, 9]. We present the case of a term infant presenting as an infarct pattern suggestive of MI in context of a thrombus in the left atrium (LA) and the results of a retrospective cohort study including all patients with a final diagnosis of myocardial infarction in the neonatal period during the last 17 years in our center.

## 2. Case Presentation

This is a full-term newborn (39 + 2 weeks of gestational age and a birth weight of 3270 grams), which is the result of third pregnancy of a healthy 36-year-old mother. After an uncomplicated pregnancy, the baby was delivered by spontaneous vaginal delivery with Apgar scores at 1 and 5 minutes of 9 and 10, respectively. The patient was discharged home completely asymptomatic at 2 days of life with exclusive breastfeeding. At 4 days of life, he was admitted to a local hospital because of a 3-hour history of respiratory distress and distal acrocyanosis. Noninvasive respiratory support with continuous positive airway pressure was commenced, and umbilical venous catheterization was performed. Over the next several hours, the patient decompensated and became hypotensive. A heart murmur was noted on exam, so an echocardiogram was done, which showed left ventricular dysfunction, thrombus in the left atrium, and signs of pulmonary hypertension. The decision was made to transfer the patient to our hospital for further cardiology evaluation and management. On arrival, the physical examination showed a nonreassuring general state, including pale/icteric color, perioral cyanosis, and tachypnea with subcostal retractions. Capillary refill was normal. Axillary and femoral pulses were present and symmetrical. Cardiac auscultation demonstrated a grade I/VI systolic murmur heard best at the left sternal border. The lungs were clear with good air entry. The abdomen was soft, with liver edge palpable 2 cm below the costal margin. The patient was hypoactive and hypotonic, with normal fontanelle and intact primitive reflexes.

**Figure 1:**
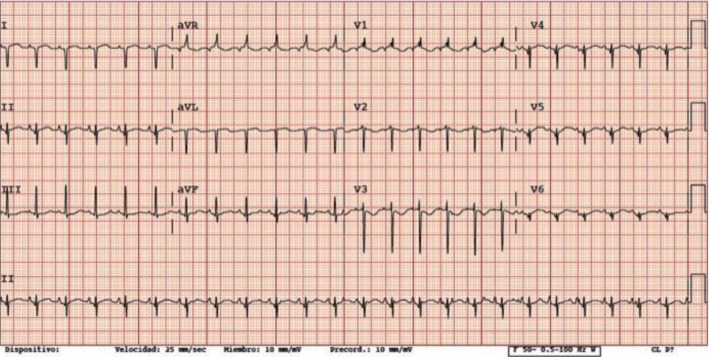
Electrocardiogram (ECG) showing a QS pattern in leads I, aVL, and V6.

Blood analysis performed at admission demonstrates moderately deranged liver function (AST 62 U/L, ALT 118 U/L, and CRP 15.3 mg/L) and markedly elevated cardiac enzymes (troponin T: 4,046 ng/L, proBNP >35,000 pg). D-dimer was 1.621 ng/mL. Chest X-ray showed cardiomegaly without pleural effusion. Electrocardiogram (ECG) showed a QS pattern in leads I, aVL, and V6 (Figure 1), and echocardiogram confirmed normal intracardiac and coronary anatomy, moderate left ventricular dysfunction (EF 45%), and a thrombus at the level of the left atrial appendage, leading to the working diagnosis of acute myocardial infarction, possibly secondary to the atrial thrombus. Hemodynamic stabilization was performed with volume expanders and milrinone infusion. Unfractionated heparin was started initially and then subsequently converted to low-molecular-weight heparin plus aspirin for full anticoagulation. Further investigations show no evidence of thrombophilia, and septic screen was negative. In follow-up echocardiograms, cardiac function showed almost complete recovery, and the patient was discharged at 26 days of age on captopril, furosemide, spironolactone, enoxaparin, and aspirin. Catheterization performed one month later did not show any lesion or abnormality in the coronary arteries, with a normal EF. Medications were gradually weaned off, and he had no further concerns.

## 3. Cohort Study

We performed a retrospective review of all patients diagnosed with myocardial infarction in our center over the last 17 years. We recorded all demographic and perinatal data including gestational age, obstetric history, Apgar score, birth weight, age at diagnosis, clinical presentation, characteristics of the electrocardiogram, troponin T values, ventricular function, angiographic study, treatment used, and mortality in each of the cases (Table 1).

A total of six newborn babies were included. The average gestational age was 36 weeks, with a birth weight of 2630 g and age at diagnosis of 4 days. Two cases included obstetric history of fetal distress, although all had a normal Apgar score. Three patients presented cardiogenic shock; the remaining presented nonspecific symptoms that were interpreted as probable sepsis. ECGs showed typical signs of infarct pattern, including characteristic appearance of Q waves and alterations of the ST segment. All patients presented with troponin elevation and cardiac dysfunction on echocardiography. An angiographic study was performed in three patients, with thickness of the coronary artery wall in only one case. The etiologies recorded in these cases were as follows: myocarditis (secondary to enterovirus) (2), congenital heart disease (global myocardial hypertrophy, aortic and pulmonary stenosis, and mitral prolapse) (1), fetal hypoxia (1), and antecedent umbilical vein catheterization (1). In one case, no cause was identified. Treatment included diuretics, inotropic support, and aspirin. Mortality rate in our series was 33%. At the time of the review, all survivors are asymptomatic, without treatment and with normal cardiac function.

## 4. Discussion

Myocardial infarction is an uncommon event in neonates compared with adults, and addressing the true incidence of this condition is difficult due to diagnostic challenges in this population. The most important causes of myocardial infarction in the neonatal period are congenital heart diseases, among which coronary vessel anomalies are the most frequent. Other reported causes include perinatal asphyxia, erythrocytosis [10], myocarditis, eosinophilic endocarditis, coagulation alterations, congenital diaphragmatic hernia, intrauterine infection, sepsis, umbilical vein catheterization, thromboembolism associated with umbilical hematoma in the renal vein or ductus venosus, and maternal diabetes during pregnancy (due to myocardial hypertrophy); congenital heart abnormalities are other possible causes. The most relevant predisposing factors are maternal arterial hypertension, fetal-maternal transfusion, fetal-fetal transfusion, and dystocic delivery [1, 11]. A significant number of cases remain classified as idiopathic [1, 2, 9].

We present a small series of infants with infarct pattern, in which myocarditis and structural heart disease were the most prominent underlying diagnoses. In the case that motivated this revision, an LA thrombus was identified presumably as a cause of the myocardium ischemia, but a coronary angiography was not performed because of the critical condition of the newborn, so it is not possible to rule out completely other causes that can mimic an MI. In some neonates and infants with underlying cardiac disease who are homozygous for the 4G/4G variant of the PAI-1 promotor polymorphism, there is a high risk of developing early thromboembolism during cardiac catheterization or with insertion of central venous lines [12], but this variant was not found in our patient.

The presentation of MI in neonates is nonspecific and may include respiratory distress, vomiting, feeding difficulties, cyanosis (with or without an oxygen requirement), tachycardia, cardiomegaly, or cardiogenic shock [9]. In our case, the clinical symptoms in combination with the echocardiographic image led us to suspect acute myocardial infarction.

The diagnosis of acute myocardial infarction in the neonatal period is based on three fundamental pillars: elevated cardiac injury biomarkers (troponin T, NT-proBNP, and CK-MB); typical ECG alterations (ST segment changes, T wave inversions, characteristic Q waves appearance, and cQT lengthening); and abnormal findings on echocardiography that include hypokinesia or akinesia of the affected area, increased echogenicity of the mitral papillary muscles, and decreased ejection fraction [1]. All of these signs were found in the cohort of patients reviewed.

Coronary angiography is the gold standard for diagnosing infarct in the adult population. However, it is not performed routinely given its potential side effects in newborns (especially in premature infants for the small caliber of blood vessels), so the risks should be assessed very carefully, as they normally outweigh the potential benefits [9].

In our patient, we decided not to perform an urgent catheterization due to clinical instability, preventing us to establish a definitive diagnosis of MI. According to the literature, other less invasive diagnostic methods can be used such as CT and MRI, but the use of ionizing radiation and iodinated contrast agent in catheter angiography is another concern particularly in children. Therefore, it should be reserved for interventional procedures or when noninvasive diagnostic imaging is inconclusive. CT and MRI play a key role in overcoming the diagnostic challenges in coronary artery imaging in children by avoiding the diagnostic pitfalls of echocardiography and reducing risks related to catheter coronary angiography. Furthermore, because it is a 2-dimensional projection imaging, catheter angiography lacks the 3-dimensional spatial relationship between the coronary arteries and adjacent cardiovascular structures. Because echocardiographic findings may be often inconclusive, CT or MRI can be used for identifying ALCAPA and the extent of myocardial infarction. Coronary CT angiography is particularly useful in newborns and young infants [13]. We currently have clinical guides that detailed comparative information of the most recent CT scanners [14].

According to the latest treatment recommendations [1], hemodynamic stabilization is recommended, and congestive heart failure should be avoided by means of beta-blockers, inotropic agents, angiotensin-converting enzyme inhibitors, diuretics, vasodilators, and calcium antagonists. If these measures are not successful, ECMO should be considered [4, 15]. Anticoagulation is the main long-term treatment. In the acute phase, unfractionated heparin should be administered in order to achieve an appropriate level of anticoagulation.

In the case of enoxaparin, we use the antixa with 0.5–1 levels, and in the case of aspirin, we use PFA-100 (collagen/epinephrine and collagen/ADP). The typical pattern in patients treated with aspirin properly is an extension of the collagen/epinephrine closure time (normal value: 73–175 seconds) with a normal collagen/ADP closure time (normal value: 50–112 seconds) [16]. Subsequently, treatment with low-molecular-weight heparin, which has fewer interactions and offers more stability, should be administered, continuing with aspirin for 1-2 years or until the resolution of the disease. P2Y receptor antagonists on platelets can be added in cases of severe ischemia. An alternative to the previous treatment would be the local administration of recombined tissue plasminogen activator (r-TPA), either locally or by intravenous continuous infusion [1, 17]. Of note, there is not enough evidence available in neonatal population to recommend generalized treatment with streptokinase or urokinase [1]. Levosimendan is a new inodilator agent that has been used in selected cases, promoting improvement of left ventricular dysfunction and increase in cardiac index [18]. Early diagnosis is the key for adequate treatment and to promote coronary reperfusion, as these patients often lack adequate collateral circulation [17]. Reversal of cardiac injury after severe myocardial infarction has been described, indicating the potential for neonatal cardiac myocytes to recover after severe injury [8]. Our series shows a high incidence of recovery with low mortality compared to other studies. We hypothesize that this could be related to smaller infarctions in distal or single branch distributions in our population.

Surgical treatment can be considered in selected cases [4], but given the potential severe complications, this approach must be individualized and carefully considered.

## 5. Conclusions

Acute myocardial infarction is an infrequent entity with a potentially poor prognosis, so a high index of clinical suspicion is needed in order to establish early and appropriate treatment. The present case attests to the importance of suspecting this diagnosis in neonates with acute cardiovascular collapse.

Precise diagnostic criteria for neonatal myocardial infarction are lacking, and the initial presentation may consist of nonspecific symptoms and signs that are commonly found in a host of neonatal disorders. Cardiac injury markers and typical ECG and echocardiogram findings are key to determine a proper diagnosis.

The most frequent treatments used in our series were diuretics, inotropic support, and aspirin. In survivors, quality of life is comparable to healthy peers due to the great capacity for myocardial regeneration. More studies are needed to provide a consensus guideline ultimately aiming to modify the prognosis of this serious disease, as well; it is worth noting that studies allowing newborns to benefit from the use of tissue plasminogen activator widely used in adults are urgently needed.

## Figures and Tables

**Table 1:** Demographic and perinatal data.

Case and year	Gestational age	Birth weight (g)	Age at diagnosis (days of life)	Clinical presentation	Ventricular function	Angiographic study	Etiology	Treatment	Mortality
7 (2002)	38	2820	14	Sepsis	Dysfunction and hypokinesia Lateral basal and lateroapical	Unrealized	Myocarditis enterovirus	Diuretic Immunoglobulins	No

2 (2007)	38	3190	1	Shock/arrythmia	Dysfunction and posterolateral hypochinesia	Normal	Fetal hypoxia	ACE inhibitor Enoxaparin ASA	No

3 (2010)	34	1720	8	Shock	Dysfunction and posterolateral hypochinesia	Unrealized	Myocarditis enterovirus (necropsy)	Immunoglobulins Diuretic Inotropic	Yes

4 (2011)	30	1700	1	Shock	Anterolateral hypochinesia	Normal	Umbilical venous channeling	ACE inhibitor Enoxaparin ASA	No

5 (2013)	40	3090	1	Heart murmur	Global myocardial hypertrophy Aortic and pulmonary stenosis Mitral prolapse	Coronary wall Hypertrophic Myocardial infarction in VI (necropsy)	Congenital heart disease	Diuretic	Yes

6 (2017)	39	3270	4	Respiratory distress and acrocyanosis	Moderate /severe Dysfunction VI Lateral/posterior and apical hypokinesia Thrombus in left atrial appendage	Unrealized	Umbilical venous channeling	Diuretic Inotropic ACE inhibitor Heparin ASA	No
