# Corrigendum to “Myocardial Infarction in Neonates: A Diagnostic and Therapeutic Challenge”

**DOI:** 10.1155/2023/9871021

**Published:** 2023-10-11

**Authors:** Manuel Rodríguez Martínez, Eladio Ruiz González, Anna Parra-Llorca, Máximo Vento Torres, Marta Aguar Carrascosa

**Affiliations:** ^1^University and Polytechnic Hospital La Fe, Valencia, Spain; ^2^Division of Cardiology, University and Polytechnic Hospital La Fe, Valencia, Spain; ^3^Neonatal Research Group, Health Research Institute La Fe, Valencia, Spain; ^4^Division of Neonatology, University and Polytechnic Hospital La Fe, Valencia, Spain

In the article titled “Myocardial Infarction in Neonates: A Diagnostic and Therapeutic Challenge” [1], the author has requested to add the following funding statement. The correct statement is as follows:
